# Co-translational profiling in the cardiac endothelium in response to LPS-induced inflammation in female mice *in vivo*: a proof-of-concept approach

**DOI:** 10.1007/s11010-026-05551-9

**Published:** 2026-04-28

**Authors:** Chad M. Warren, Bhairavi Swaminathan, Paulina Langa, Stephanie R. Villa, Walter C. Thompson, Magdalena Chrzanowska, Jan K. Kitajewski, R. John Solaro, Beata M. Wolska, Paul H. Goldspink

**Affiliations:** 1 Department of Physiology and Biophysics, University of Illinois, Chicago, IL USA; 2 Versiti Blood Research Institute, Milwaukee, WI USA; 3 Department of Pharmacology and Toxicology, Medical College of Wisconsin, Milwaukee, WI USA; 4 Cancer Center, Medical College of Wisconsin, Milwaukee, WI USA; 5 Cardiovascular Center, Medical College of Wisconsin, Milwaukee, WI USA; 6 Center for Cardiovascular Research, University of Illinois, Chicago, IL USA; 7 University of Illinois Cancer Center, University of Illinois, Chicago, IL USA; 8 Department of Medicine, Division of Cardiology, University of Illinois, Chicago, IL USA; 9 Department of Physiology and Biophysics (M/C 901), College of Medicine, University of Illinois, 1853 W. Polk St., Room 522, Chicago, IL 60612 USA

**Keywords:** Heart, Endothelium, Translatome, Ribosome, Lipopolysaccharide

## Abstract

Producing functional proteins involves multiple steps during mRNA translation on the ribosomes. However, co-translational regulatory mechanisms remain poorly characterized in intact mammalian systems. As a proof-of-concept, we developed a multi-omics approach to investigate endothelial-specific, co-translational regulation by modifying the translating ribosome affinity purification (TRAP) in vivo. We simultaneously co-immunoprecipitated (IP) polysome-associated mRNAs and proteins from the hearts of hemagglutinin-tagged ribosomal protein L22 mice (RiboTag) crossed with inducible endothelial-specific *Cdh5CreERT2* mice (*RiboTag*_*EC*_). To perturb endothelial function, female mice were injected with E. *coli* lipopolysaccharide (LPS) (6 mg/Kg, i.p., 12 h). Hearts were homogenized, with ~ 10% used for input RNA-Seq and proteomics controls, and the remainder for IP of ribosome-bound polyadenylated mRNA and proteins. Endothelial cell transcripts (*pecam1*, *cdh5*) were enriched > 5-fold, while markers characteristic of other cell types were significantly depleted (< 0.05 q-value). We aligned transcriptomic and proteomic datasets (> 1250 overlapping terms) to identify pathways associated with concordant and discordant co-translational regulation. LPS was identified as the upstream regulator of the co-translational dataset that was concordantly regulated. Upregulated mRNAs but not proteins related to glycolysis were discordantly regulated. These findings validate our proof-of-concept multi-omics approach as a predictive platform for identifying disease-relevant pathways regulated at the co-translational level *in vivo.*

**Supplementary Information:**

The online version contains supplementary material available at 10.1007/s11010-026-05551-9.

## Introduction

Proteins serve as functional, structural, and regulatory molecules in cells, playing an essential role in the homeostasis of tissues and the complex physiological functions of organs. The regulation of protein production is initiated by key processes coupling messenger RNA translation with nascent polypeptide maturation on the ribosome. During normal growth of eukaryotic cells, approximately 5 million ribosomes translate mRNAs to produce proteins at a rate of roughly 10^6^ proteins per minute [1].

Tools that enable the investigation of molecular regulation of DNA and changes in the composition of cell-specific mRNA and proteins are central to understanding the complex interactions in health and disease in both model organisms and humans. Gene expression analysis is a powerful approach that can provide insight into how genes respond to extracellular signals and their cellular function. Because gene expression is precisely regulated, it can act as an “on/off switch” and a rheostat that fine-tunes protein levels in different cell types and conditions. Over the last decade, several technological advances have enabled the analysis of large numbers of genes, and with current RNA-Seq-based technologies, the transcriptome within individual cells can now be measured in depth [2]. Single-cell isolation and separation from complex tissues are critical steps in single-cell RNA sequencing scRNA-seq, often requiring tissue processing and specialized equipment. This technology permits quantitative analysis of transcriptomic profiles of hundreds of thousands of cells, cellular identity, and heterogeneity from complex tissues [3]. However, complementary approaches that examine and quantify the proteins translated from those transcripts remain comparatively limited and less well established.

Single-cell proteomic approaches aimed at studying the translation of proteins identified with scRNA-Seq are presently at the forefront of technological development and offer the capability to examine protein translation at remarkable resolution [4]. Despite the potential power of this approach, there are challenges associated with isolating specific cell types to sufficient purity and abundance, the inability to amplify proteins for in-depth mass spectrometry, and, if feasible, the eventual move into the in vivo environment. To date, most multi-omics studies in isolated cells have usually been performed in parallel to combine bulk transcriptomic and proteomic datasets or have developed computational tools to align datasets to provide insight into protein translation [5–10]. As such, some studies have shown a poor correlation between mRNA and protein profiles in response to extracellular signaling and injury, highlighting the different regulatory mechanisms at the transcriptomic and proteomic levels involved in the overall regulation of protein abundance [11–14].

To overcome some of these limitations, we developed a proof-of-concept multi-omics approach to investigate co-translational regulation by simultaneously analyzing the actively translating transcriptome and proteome in the endothelium of the mouse heart in vivo. We developed methods to co-immunoprecipitate polysome-associated endothelial-specific mRNA and proteins as they are translated on the polysome. Sensitive and quantitative RNAseq and proteomic data allowed us to interrogate, for the first time, the “functional translatome” in an intact mammalian organ in vivo. An advantage of studying cotranslational complexes in their physiological environment is the ability to analyze cell- or tissue-specific interactions that might not otherwise occur in vitro.

Several steps regulate which mRNAs are translated, and cellular protein abundance is mainly controlled at the ribosomal translation initiation step. The ribosomes also function as an organizing hub for chaperones and modifying enzymes that help coordinate the timing of polypeptide folding with specific regulatory protein networks to maintain proteome integrity [15–17]. Ribosomal proteomics and profiling (Ribo-Seq) have provided significant insight into ribosomal protein diversity and ribosomal regulation of translation and co-translational events. Within the context of these studies, it is recognized that ribosomal protein heterogeneity and ribosome-associated proteins can play vital roles in regulating the translation of different mRNAs in cells from various tissues [18, 19]. Advances in ribosomal profiling techniques, which capture mRNA bound to ribosomes to identify mRNA-protected fragments, have enabled the study of translation kinetics and the inference of protein translation in animal models and human disease in vivo [20, 21]. Even though these studies have enabled analysis of specific cell types within complex tissues, the roles of ribosomal composition and the translation of signaling proteins and networks in response to physiological and pathophysiologic changes in an organism remain largely unknown. This gap primarily stems from a paucity of approaches for simultaneously isolating cell-specific ribosomes, mRNAs, and proteins from tissues for quantitative analysis in vivo. Consequently, we hypothesized that the ribosomal protein epitope-tagged transgenic reporter mouse (RiboTag) could enable spatial regulation of protein production at the polyribosome/mRNA complex in vivo. This model permits crosses with cell-specific inducible Cre-recombinase driver lines and was initially developed to facilitate the isolation of cell-specific translating mRNAs on the polyribosomes in vivo [22]. Thus, in this proof-of-concept study, we adapted this technology to isolate and quantify proteins within the polyribosome/mRNA complex in heart endothelial cells in response to inflammatory stress *in vivo.* Our data demonstrated that transcripts and proteins were concordantly regulated within our datasets, enabling us to identify changes in endothelial-specific signaling pathways in response to a known stimulus by analyzing actively co-translating transcript and protein networks in *vivo.* Moreover, transcripts and proteins that are discordantly regulated within our datasets indicate co-translational control of cellular processes that would not be evident with transcriptomic analysis alone.

## Materials and methods

### RiboTag_EC_ mouse model

**Fig. 1:**
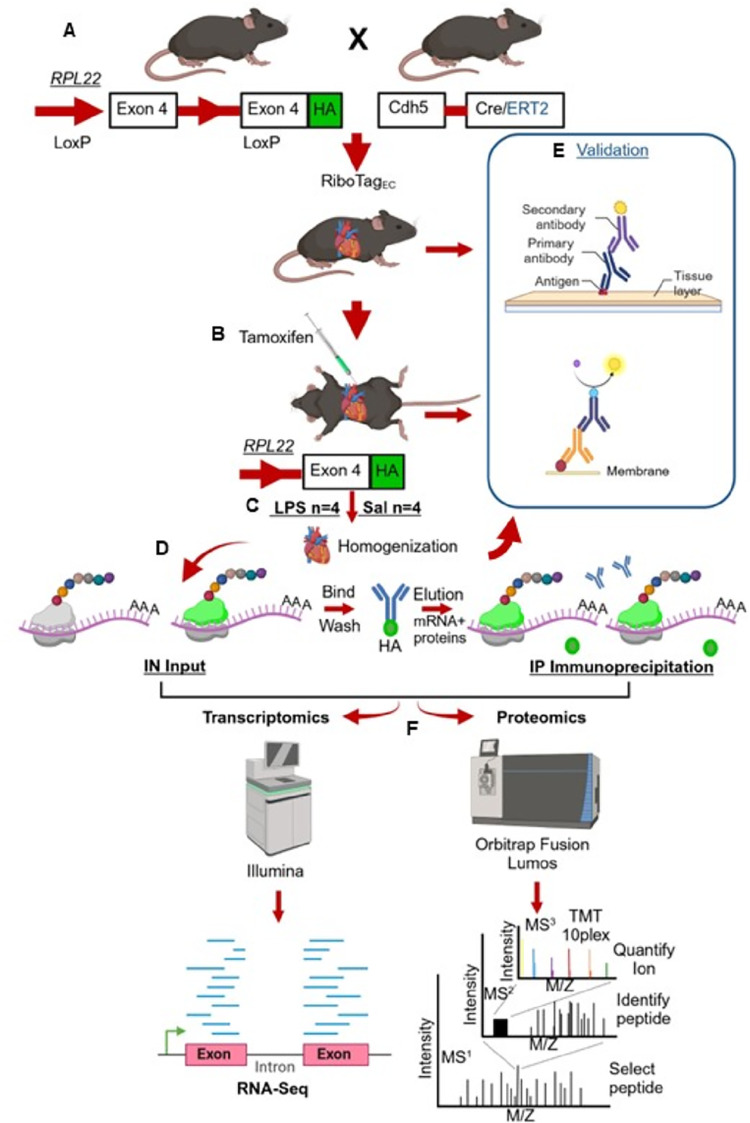
Workflow diagram of the “functional translatome” discovery approach. **A**, The HA-tagged ribosomal protein L22 (RPL22) reporter mouse (RiboTag) was crossed with a tamoxifen-inducible endothelial-specific Cdh5-CreERT2 mouse (RiboTag_EC_), allowing simultaneous endothelial-specific immunoprecipitation (IP) of mRNA and nascent proteins. **B**, Tamoxifen was injected intraperitoneally with a dose of 200 mg/kg [27] for 5 consecutive days, beginning at 3–4 weeks of age, to induce Cre recombination. **C**, The mice were injected with lipopolysaccharide (LPS) (6 mg/Kg [30], i.p. 12 h) or saline (Sal) to determine the effects of LPS. **D**, The excised mouse hearts were homogenized and processed for input (IN) and IP samples. **E**, Additional samples were collected for validation by immunohistochemistry and Western blot analysis. **F**, Four biological replicates were then analyzed by RNA-Seq and quantitative proteomics. Created with BioRender.com

The IACUC of the University of Illinois, Chicago, approved all animal protocols (Protocol #18 − 012), which also conforms to the US Public Health Service Policy on Humane Care and Use of Laboratory Animals. The reported methods comply with the ARRIVE guidelines (https://arriveguidelines.org/arrive-guidelines). RiboTag (*Rpl22*^*HA/+*^*)* [22] mice were kindly provided to the Kitajewski lab for use by Peter Canoll (Columbia University, New York City, NY). Endothelial-specific VE-cadherin-Cre mice (*Cdh5*^*CreERT2*^) [23, 24] were developed by Ralf Adams Laboratory at the London Research Institute and obtained under an MTA with Cancer Research Technology Limited, UK, with Drs. Kitajewski and Goldspink. The resulting *Cdh5-Cre*^*Tg/Tg*^*/RiboTag*^*flox/flox*^ (RiboTag_EC_) cross (Fig. 1) was maintained in a C57BL6/J background as previously described [25, 26]. Tamoxifen was prepared in corn oil at 20 mg/ml and injected intraperitoneally at 200 mg/kg for 5 consecutive days, beginning at 3–4 weeks of age, to induce Cre recombination [27]. No adverse effects of tamoxifen were found in any animals post-injections, so all animals were included in the study. All animals in this study were females and 4–5 months old at the time of the experiment. For this proof-of-concept study, we chose to use females to improve survival outcomes following LPS treatment, as estrogen is known to exert vascular-protective and immune-modulatory effects [28, 29], thereby enabling a more reproducible approach with fewer mice. The multi-omic approach used a biological *n* = 4 per group, and the animal group assignment was randomized via https://www.graphpad.com/quickcalcs/randomize1/. One group received saline (Sal); the other received 6 mg/kg [30] *E. coli* O111:B4 bacterial lipopolysaccharide (LPS) from Sigma-Aldrich (Cat # L2630), and all were injected intraperitoneally. Our experimental study design excluded animals that died during LPS or saline administration or did not survive the 12 h incubation; however, no exclusions were necessary. The study design was a binary comparison between LPS and saline in endothelial-enriched (IP, immunoprecipitation) heart tissue or in non-enriched whole-heart homogenate (IN, input). The four binary comparisons were IP LPS vs. IP Sal, IN LPS vs. IN Sal, IP Sal vs. IN Sal, and IP LPS vs. IN LPS. Mice were euthanized 12 h after LPS or saline injection by anesthetizing with Ketamine (200 mg) + Xylazine 20 mg/kg via IP. When an adequate depth of anesthesia was attained, as determined by the absence of the toe-pinch reflex, mice were euthanized by cervical dislocation. A cardiectomy was performed, and the heart was rinsed in ice-cold saline, flash-frozen in liquid nitrogen, and stored at −80 °C until use. Additional animal hearts were prepared as described for the Western blot and immunohistochemical (IHC) validation experiments described below. A female wild-type C57BL6/J mouse heart was a negative control and was prepared for proteomics to account for non-specific binding to the HA-bound Dynabeads described below.

### Multi-omics ribosome immunoprecipitation (IP) for transcriptomics and proteomics preparation

Samples were prepared concurrently for transcriptomics and proteomics from the same heart. Dynabeads from Life Technologies (cat # 10002D) were used to immunoprecipitate (IP) the HA-tagged ribosomal protein L22 from coronary endothelial cells. The Dynabeads (50 µl of slurry) were prepared by removing the storage buffer and washing the beads two times in 100 µl each of IP wash buffer (mM): 20 Tris-HCl, 250 NaCl, 15 MgCl_2_, 100 µg/ml cycloheximide, 0.5% (v/v) Triton X-100, 0.16U/µl SUPERase RNase inhibitor (Invitrogen #AM2696), 0.012U/µl Turbo DNase (Invitrogen #AM2238), 0.5X Halt protease inhibitor and 0.5X EDTA (Thermo Scientific #78430). A rabbit-anti-HA-antibody (Abcam # ab9110) at 1 µg/µl was diluted to 0.05 µg/µl in a total of 100 µl of IP binding buffer (mM): 20 Tris-HCl, 250 NaCl, 15 MgCl_2_, 100 µg/ml cycloheximide, 0.5% (v/v) Triton X-100. The diluted antibody was then added to the washed Dynabeads and incubated at room temperature for 30 min with continuous, gentle mixing. The diluted antibody was then separated, and the beads were washed once with 100 µl of IP binding buffer, stored in 100 µl of IP binding buffer at 4 °C, and used within 24 h.

The frozen hearts (~ 100 mg) were homogenized in lysis buffer (mM): 20 Tris-HCl, 150 NaCl, 15 MgCl_2_, 100 µg/ml cycloheximide, 1% (v/v) Triton X-100, 1 DTT, 0.3U/µl SUPERase RNase inhihitor (Invitrogen #AM2696), 0.024U/µl Turbo DNase (Invitrogen #AM2238), 1X Halt protease inhibitor and 1X EDTA (Thermo Scientific #78430) at a 1:10 tissue: buffer ratio on wet ice with a Duall homogenizer. The samples were spin clarified at 9,400 X g for 10 min at 4 °C, and 10% of the total supernatant was split into two tubes as input (IN) for transcriptomics and proteomics. The remaining supernatant (90% v/v) was divided in half and added to two tubes as IP for transcriptomics and proteomics using HA-bound Dynabeads, and incubated with gentle mixing for 2 h at 4 °C. The sample was magnetically separated from the Dynabeads and washed 3 times with 800 µl IP wash buffer. The washed Dynabeads were then eluted for transcriptomic and proteomic analyses, as described below.

### RNA preparation, sequencing, and data processing

Immunoprecipitated RNA was purified from the Dynabeads with an RNeasy Plus Mini Kit (Qiagen cat #74134) following the manufacturer’s protocol with minor modifications. Washed beads were eluted twice for 5 min each at room temperature with 300 µl of RLT with β-mercaptoethanol. The IN samples were diluted (at least 10-fold) to a final volume of 600 µl at room temperature with RLT buffer plus β-mercaptoethanol. One additional 500 µl wash with RPE buffer was added to the protocol, along with a 10 min room-temperature incubation with 40 µl RNase-free water, which was added to the center of the spin column, centrifuged, and then reapplied to elute the RNA. The purified RNA was quantified and quality-checked using NanoDrop, Qubit, and an Agilent 4200 tape station (all RIN values > 8.5) at the Genomics Research Core within the Research Resources Center at the University of Illinois, Chicago. A total of 16 samples were sequenced at a depth of ~ 20 million 150-base paired-end reads using an Illumina NovaSeq 6000 PE150 from the services of Novogene Corporation. The 16 samples comprised four experimental groups: IP Sal, IP LPS, IN Sal, and IN LPS, with a biological *n* = 4 in each group. The IN grouping represents the total mRNA from the whole heart extract, while the IP group is the ribosome-bound mRNA specific to the endothelium. The reads were mapped to the mouse transcriptome UCSC/mm10 using STAR aligner (v. 2.5.2b) [31] and processed with Samtools (v. 1.4.1). The bam files were processed using FeatureCounts [32] (Subread package, v.1.5.2) to obtain raw counts. DESeq2 [33] (v. 1.18.1) was used to identify differentially expressed genes, and GraphPad Prism 10.2.2.

### Protein peptide generation

Nascent proteins were purified from the Dynabeads by eluting with 30 µl of IP elution buffer (mM): 5% (w/v) SDS, 20 DTT, 8 M urea, 100 Glycine, pH 7.55, and incubating for 5 min at 95 °C, then re-applying the elution buffer and repeating once. The IN samples were diluted with an equal volume of 10% (W/V) SDS, 40 mM DTT. The concentration of the samples was determined with a Pierce 660 nm protein assay (Pierce #22660) with the addition of the ionic detergent compatibility reagent following the manufacturer’s protocol. All samples were diluted with IP elution buffer to no more than 80 µg of protein in 25 µl and incubated for 20 min at 30 °C to reduce. The samples were then alkylated with 40 mM iodoacetamide for 30 min at room temperature in the dark. S-TRAP micro spin columns (Protifi #C02-micro-80) were used as directed by the manufacturer’s standard protocol with minor modifications to digest the samples. The peptides were digested with Trypsin/Lys-C (Promega #V5071) at a 1:10 enzyme: protein ratio in 50 mM triethylammonium bicarbonate (TEAB), pH 8.5, for 1 h at 47 °C in a thermomixer without shaking. The peptides were eluted according to the manufacturer’s protocol, with the elution step repeated once, and two eluates pooled. The pooled peptide eluates were evaporated to dryness using a SpeedVac and stored at −80 °C.

### Isobaric TMT labeling and high-pH-reverse-phase fractionation

The pooled peptide samples were resuspended in 50 µl of 50 mM TEAB pH 8.5 and 8 M urea, and the peptide concentration was determined using Pierce’s Fluorometric peptide assay (#23290) following the manufacturer’s recommendations. All peptide samples were diluted to the same concentration, and ~ 16% (v/v) was removed from each sample and equally mixed to serve as an internal global standard. The peptide samples (2.6 µg each) were randomly isobarically labeled to minimize labeling bias with Thermo Fisher Scientific’s TMT (Tandem Mass Tag) 10-plex reagents (cat# 90110; lot# TJ268160) following the manufacturer’s recommendations, with all volumes proportionally scaled to the amount of peptide labeled. The labeled and quenched samples were then equally mixed into two separate experiments (1 + 2) due to the number of samples. The non-transgenic negative control hearts did not yield enough protein following IP to TMT label compared to the RiboTag samples, making the comparison invalid. Consequently, they were not included in the quantitation (TMT channel 131 experiments 1 + 2) but were mixed with the other TMT channels, indicating that the immunoprecipitation was successful. The internal global standard was labeled with the TMT126-channel for both experiments. The two separate experiments were evaporated to dryness using a SpeedVac and stored at −80 °C until the high-pH reverse-phase fractionation was performed.

The TMT-labeled peptides were resuspended in 300 µl of 1.5% (v/v) trifluoracetic acid (TFA) to clean and fractionate with Pierce’s high-pH-reverse-phase kit (cat# 84868) with modifications previously described [34]. The 12 fractions from each experiment were evaporated using a SpeedVac and resuspended in 25 µl of 25% (v/v) acetonitrile and 0.1% formic acid. The concentration of the peptides was determined using Pierce’s colorimetric peptide assay (Cat# 23275) according to the manufacturer’s protocol. The 12 fractions from each experiment were then evaporated using a SpeedVac, and the amount of peptide was recorded for each fraction. The fractions were then sent to the UC-Davis Proteomics Core Facility to run in a Thermo Scientific Orbitrap Fusion Lumos.

### Mass spectrometric data acquisition

The peptide fractions were resuspended in 2% (v/v) acetonitrile and 0.1% (v/v) formic acid. 800 ng of each peptide fraction was injected in 6 µl sequentially with an Ultimate 3000 RSLCnano UHPLC in conjunction with an EASY-Spray source operating in the positive ion mode into the Thermo Scientific Orbitrap Fusion Lumos. A Thermo Scientific Acclaim PepMap 100 C18 reversed-phase column (DX164199, 100 μm X 20 mm, 100 Å, 5 μm) trapped the peptides, and the separating column was a C18 reverse-phase Thermo Scientific column (ES802, 75 μm X 250 mm, 100 Å, 2 μm). The peptides were eluted with an acetonitrile gradient from 2 to 50% over 180 min with a flow rate of 200 nl/min at 40 °C. Data were acquired on the Orbitrap Fusion Lumos using the parameters as previously described, with minor modifications [34, 35]. An Orbitrap survey scan was performed from 350 to 1600 m/z with a resolution of 120 K and a target of 5 × 10^5^ ions or a maximum injection time of 50 ms. Data-dependent MS/MS spectra were acquired in the linear ion trap operating in turbo mode, using collision-induced dissociation at 35% energy and an activation Q of 0.25. The MS/MS spectra were acquired with a target of 1 × 10^4^ ions or a maximum of 50 ms, an isolation mass window of 0.7 m/z, charge states 2–7, and a 60 s dynamic exclusion. Synchronous precursor selection with up to 10 precursors was selected for MS3 with higher-energy collisional dissociation at 65% energy, a mass range of 100–500 m/z, resolution of 50,000, and a target of 1 × 10^5^ ions or a maximum injection time of 105 ms. A precursor selection range of 400–1200 m/z was used along with an isolation window of 2 m/z and a fixed cycle time of 3s.

### Mass spectrometric analysis and TMT quantification

Thermo raw data files were obtained from the UC-Davis Proteomics core, and the data were searched using PEAKS Studio v11.5 build 20,230,821 (Bioinformatics Solutions Inc.). Database search parameters were conducted with a precursor mass tolerance of 10 ppm, a fragment mass tolerance of 0.5 Da, and trypsin specified as the proteolytic enzyme, allowing up to two missed cleavages. One fixed modification (TMT10plex + 229.16) and three variable modifications (carbamidomethylation + 57.02, carbamylation + 43.01, oxidation M + 15.99) were specified with a maximum of 3 variable modifications per peptide. The database alignment was performed against a reviewed mouse UniProtKB Swiss-Prot database (downloaded on Aug 8th, 2023), with 17,735 entries using a decoy-fusion approach, allowing for a more conservative false discovery rate (FDR) estimation [36]. A contaminant database cRAP (downloaded March 4th, 2019), from www.thegpm.org/crap/ was used for a contaminant database. The deep learning boost was applied alongside peptide and protein group FDR cutoffs of 1% and 5%, respectively. Positive protein identification required at least 2 unique peptides. For data refinement, the chimera association was set to false, while mass correction was enabled.

The initial TMT quantification was first performed in PEAKS Studio v11.5 build 20,230,821 (Bioinformatics Solutions Inc.). Normalized data were exported to an Excel file and statistically analyzed by GraphPad Prism 10.2.2 and OriginPro 2024 (64-bit) SR1. The initial PEAKS Studio analysis was conducted with a mass tolerance of 10 ppm on the MS3 reporter ion without purity correction. Both intra- and inter-normalization were performed utilizing the total ion counts method, and the modified peptide forms were excluded from quantification. Inter-experimental normalization was performed using a global internal standard (TMT channel 126) to link the two TMT-labeled experiments. In each binary comparison (IP LPS vs. IP Sal, IN LPS vs. IN Sal, IP Sal vs. IN Sal, and IP LPS vs. IN LPS), a reference label was assigned in PEAKS Studio for intra-auto-normalization and ratio purposes. The reference label for each binary comparison was chosen based on the appropriate control group (input or saline) and the TMT channel with the highest number of peptide identifications. Reference channel proteins were required for all comparisons. In each case, the reference labels were derived from experiment 2, with the following TMT channels assigned: 128 N for IP LPS vs. IN LPS; 129 C for IP LPS vs. IP Sal; 130 C for IN LPS vs. IN Sal, and IP Sal vs. IN Sal. The normalized data was exported to an Excel document for further analysis, as described in the statistical analysis section below.

### Immunohistochemistry validation

A new set of hearts was collected as described above for immunohistochemistry validation. Hearts were rapidly sectioned at the midpapillary level, placed into embedding cassettes, and fixed in 10% neutral buffered formalin (Milipore-Sigma, HT501128). Samples were then washed and stored in 70% (v/v) Ethanol. Fixed samples were paraffin-embedded, and non-consecutive transverse sections were cut and mounted onto microscope slides (Research Histology Core, UIC). Slides were baked at 60 °C for 20 min and deparaffinized in 100% xylene (2 × 7 min), followed by rehydration through a graded ethanol series (100% for 2 × 5 min, 95% for 5 min, 70% for 5 min, and 50% for 5 min). Sections were then rinsed in distilled water for 20 min. Antigen retrieval was performed using Tris-EDTA buffer (mM): 10 Tris-base, 1 EDTA, 0.05% (v/v) Tween 20, pH 9.0 at 95 °C for 90 min [37, 38].

Slides were blocked in 5% BSA in phosphate-buffered saline (PBS, Corning # 46-013-CM) 0.1% (v/v) Tween 20 (PBST) for 1 h at room temperature. To label the endothelium, slides were incubated in rat monoclonal anti-CD31 antibody (1:10, cat. DIA-310, Dianova), and to detect the HA epitope, a rabbit polyclonal anti-HA antibody (1:100, cat. ab9110) was used with both antibodies diluted in 1% BSA PBST and incubated overnight at 4 °C. Slides were washed three times, 5 min each with PBST, and then incubated for 2 h at room temperature with secondary antibodies diluted to 1:500 in 1% (w/v) BSA in PBST: donkey anti-rat Alexa Fluor 594-conjugate (ThermoFisher cat# A21209) for CD31 and chicken anti-rabbit Alexa Fluor 488-conjugate (ThermoFisher cat# A21441) for HA. Slides were washed three times for 5 min in PBST and incubated with DAPI (4’,6-diamidino-2-phenylindole) for 20 min at room temperature for nuclear counterstaining. Slides were washed in PBST and mounted with a mounting medium that preserves fluorescent signal (Thermo Fisher Scientific, P10144).

All slides were imaged using a confocal microscope Zeiss LSM880 (Germany) fitted with a C-Apochromat 40x/1.2 W Korr FCS M27 objective. Airyscan imaging was performed at a resolution of 1024 × 1024 pixels with 16-bit depth, focusing on the regions of interest. Standard confocal acquisition was performed using a GaAsP photomultiplier tube with a Quasar detector (GaAsP), flanked by multi-alkali PMTs. Emission light was filtered through emission filters (EF5), and signal intensity was modulated using acousto-optical tunable filters to optimize brightness. Fixed slides were imaged at room temperature with ZEISS Black 2.3 SP1 acquisition software, and image analysis was performed with ZEISS Blue edition 3.2. Fluorochromes and their respective excitation/emission maxima were as follows: Alexa Fluor 488 (494 nm/517nm), Alexa Fluor 561 (558 nm/575nm), and 4′,6-Diamidino-2-phenylindole (350 nm/465 nm). The beam splitters used were MBS 488/561/633, MS INVis MBS-405, and DBS1: SBS LP 660.

### Immunoblot validation

An additional cohort of hearts was collected and homogenized as described above; however, instead of enzymatic digestion, the samples were diluted in 8 M urea, 5% (w/v) SDS, 100 mM glycine, pH 5.8, and 50 mM β-mercaptoethanol, then separated by 15% SDS-PAGE gel, as previously described [30]. Proteins were transferred to nitrocellulose 0.2µ membrane in 10 mM CAPS, pH 11.0 [39], at 20 V for 90 min in a Criterion transfer cell. The membrane was stained with Swift stain (G Biosciences, Cat# 786 − 677) according to the manufacturer’s instructions and used as a qualitative loading control. The immunoblot was blocked in 5% (w/v) non-fat dry milk diluted in Tris-buffer saline (50 mM Tris-base pH 7.5, 200 mM NaCl) with 0.01% (v/v) Tween-20 (TBST). The membrane was incubated overnight at 4 °C with a rabbit anti-HA primary antibody (Abcam # ab9110) diluted 1:10,000 in blocking buffer. Following three 5 min washes with TBST, the membrane was incubated for 90 min at room temperature with an HRP-conjugated anti-rabbit secondary antibody (Cell Signaling # 5127) diluted 1:20,000 in blocking buffer. After 3 additional washes with TBST, signal was developed using SuperSignal West Femto chemiluminescent substrate (ThermoScientific Cat# 34096) and imaged with a Chemidoc MP system (BioRad) using Image Lab v 6.1.0 software for acquisition and analysis.

### Statistical analysis

RNA-seq data were exported from DESeq2 into GraphPad Prism 10.2.2 for log_2_ transformation and statistical analysis using an unpaired t-test, assuming individual variances for each row. Multiple comparisons were calculated using the false discovery rate, utilizing a two-stage linear step-up method [40] with a Q-value and P-value set at 0.05 for significance. The same analytical workflow was applied to normalized proteomics data exported from PEAKS Studio for a consistent analysis between the RNAseq and proteomic data sets. Data distribution for both datasets was assessed in GraphPad Prism by Skewness and Kurtosis, confirming normal distribution across all samples. Intensity or count data were imported into OriginPro 2024 (64-bit) SR1 for principal component analysis (PCA). No statistical analysis was performed for the qualitative IHC or Western blotting experiments.

### Bioinformatics

The transcriptomic and proteomic data were aligned in Excel based on concordant and discordant expression changes. Datasets from both the input (whole heart) and IP (endothelial) fractions - including gene names, Uniprot identifiers, Log2 ratios, P-values, and Q-values - were imported into Ingenuity Pathway Analysis (IPA) for core analysis [41] and comparison analysis (transcriptomics *vs* proteomics). Core analysis was performed to identify direct and indirect relationships between genes and endogenous chemicals using experimental log-ratios, p-values, and false discovery rates (q-values). IPA uses right-tailed Fisher’s exact test to calculate P-values and determine the significance of enrichment for canonical pathways and upstream regulators, reflecting associations between the experimental dataset and the curated reference database. Complete proteomic datasets for IP LPS *vs*. IP Sal and IN LPS *vs*. IN Sal comparisons were imported into IPA separately, applying a ≥ 20% difference cutoff. Similarly, complete transcriptomic datasets for the same binary comparisons were imported into IPA separately, using a ≥ 50% difference cutoff and significance thresholds of p-value and q-value ≤ 0.05. We performed Gene Set Enrichment Analysis (GSEA) for the list of protein abundance changes between IP LPS vs. IP Sal using the *gseGO()* function with *ont=”ALL”*, to extract terms in the *BP*, *MF*, and *CC* categories (clusterProfiler package, R) [42]. Dotplots were generated using DOSE (R package) to visualize the top 20 GO terms with the most significant adjusted P-values, representing either activated or suppressed [43].

STRING protein-protein association networks and functional enrichment analysis [44] of IP LPS vs. IP Sal discordant data (protein abundance was down while the transcript expression was upregulated) indicated the main change was in glucose metabolism. All data and downloaded files in string-db.org are freely available under a “Creative Commons BY 4.0” license. The minimum required interaction score was set to high confidence (0.700). The k-means clustering was based on their centroids and set to k = 4 “natural” clusters, where the top cluster (+ Glucose catabolism) with 24 proteins was further visualized under the KEGG pathways category and sorted by signal within STRING.

## Results and discussion

### The functional translatomics approach

The RiboTag_EC_ mouse model was initially created to investigate endothelial cell (EC)-specific transcriptomic changes in vivo (Fig. 1). Adopting a multi-omics approach by immunoprecipitating mRNA and proteins as they are being translated on the polysome enabled us to simultaneously interrogate the transcriptome and proteome to investigate the functional translatome (Fig. 1). Here, we demonstrate for the first time that endothelial-specific co-translational regulation in response to LPS can be successfully studied in vivo using a single adult mouse heart. This refined approach identified changes in endothelial-specific signaling pathways by analyzing actively co-translating transcripts and protein networks, focusing on concordant and discordant expression patterns within the translatome.

Polysome profiling, which separates translated mRNAs associated with polysomes using sucrose-gradient centrifugation, has been a foundational method and previously applied by our group in isolated cardiac myocytes [45]. Recently, advanced approaches such as ribosome profiling (ribosomal footprinting) and translating ribosome affinity purification (TRAP) have been developed to analyze translation at the cell-type level. These methods rely on sequencing ribosome-protected mRNA fragments following ribonuclease digestion of unprotected regions of RNA, generating ribosome footprints (RFPs) that reveal which transcripts are actively being translated. This enables position-specific mapping of the translating ribosomes on the mRNA, allowing the analysis of translation dynamics and kinetics, precise determination of open reading frame boundaries, start and stop sites, and qualification of translatome changes under stress and disease conditions in vitro and *vivo* [46]. Further developments of this elegant approach have shed light on ribosomal stalling and collisions, which impact translational efficiency [47, 48]. However, while these approaches examine ongoing synthesis, they do not directly assess all stages of protein translation as they do not account for protein quality control and degradation during co-translation and post-translation.

**Fig. 2:**
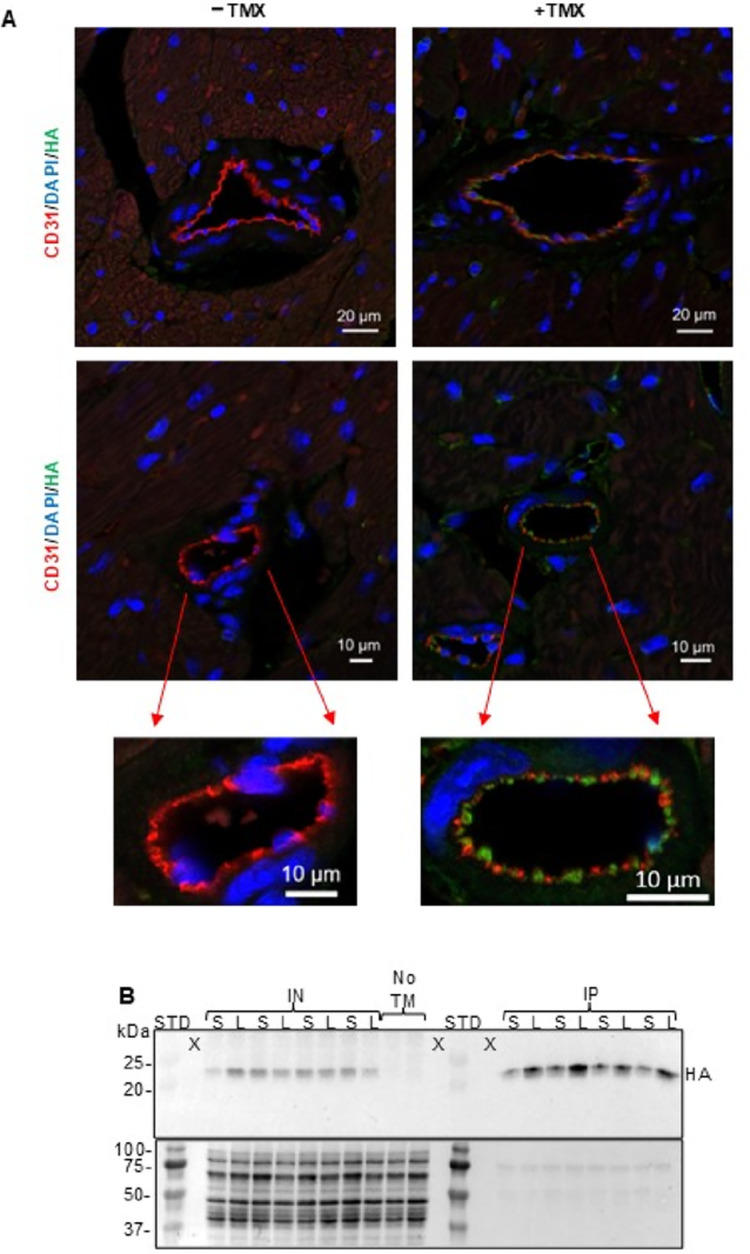
Validation of the induction of the HA-tagged ribosomal protein L22. **A**, Immunohistochemical staining of transverse heart sections with antibodies to CD31 (endothelial vessel linings, red), HA (HA-tagged RPL22, green), and DAPI (nuclear stain, blue). Tamoxifen (TMX)- injected mice + TMX show HA-tag localized to CD31-positive vessels; conversely, -TMX does not co-localize. Scale bars = 20µM (top) or 10 μm (bottom). The two lowest images are zoomed-in versions of the above at the originating arrows. **B**, An immunoblot is shown probed with an HA antibody (top) and stained with Swift stain (total protein stain) as a qualitative loading control (bottom). *In* input, *IP* immunoprecipitated, *S* saline, *L* lipopolysaccharide, *X* blank lane, *STD* molecular weight standard. Note: Animals not injected with tamoxifen (No TMX) did not show the HA-tagged RPL22 band at ~ 23 kDA

To confirm the expression and localization of the epitope-tagged large ribosomal subunit protein RL22 in cardiac endothelium, heart sections from RiboTag_EC_ mice were co-stained with antibodies against CD31 (platelet endothelial cell adhesion molecule-1), a marker of endothelial cells and some hematopoietic cells, and hemagglutinin (HA). As shown in Fig. 2A, CD31 expression was localized to the endothelial lining of the coronary vessels. In RiboTag_EC_ mice that did not receive tamoxifen (-TMX), the HA-tagged RL22 fusion protein was not detected, serving as a negative control (Fig. 2A). In contrast, tamoxifen-treated mice showed robust expression of the HA-tagged RL22 fusion protein co-localized with the CD31-positive cells. This endothelial-specific expression was further validated using an orthogonal approach. Whole heart lysates (IN) and immunoprecipitated (IP) fractions from RiboTag_EC_ mice were immunoblotted with an HA-specific antibody (Fig. 2B). Strong immunoreactive protein bands with a relative gel migration of ~ 23 kDa (HA+RPL22 molecular weight) were found in all protein samples from mice injected with tamoxifen. In contrast, no bands were visible in the -TMX negative controls (Fig. 2B). Additionally, HA immunoreactive bands in the LPS-treated IP samples appeared more intense, qualitatively suggesting an increased abundance of ribosomal proteins in response to LPS treatment. This observation was further supported by a corresponding increase in the log-ratio IP LPS/IP Sal for all ribosomal subunit proteins detected in the proteomic IP fraction following LPS treatment (Table S1). Overall, we identified 75 of the reported 80 [49] eukaryotic ribosomal protein components of the 40 S and 60 S subunits within our co-translational complexes, demonstrating we had effectively performed translating ribosome affinity purification (TRAP) following immunoprecipitation with the HA-tagged RPL22 (Table S1). All ribosomal proteins were excluded from the datasets to focus the subsequent bioinformatic analysis on functional and signaling pathways relevant to the endothelial polysome-associated proteome in response to LPS.

LPS is an endotoxin found in the outer membrane of Gram-negative bacteria and is used as an experimental agent to trigger an inflammatory response primarily by activating the Toll-like receptor (TLR)−4 sensor on macrophages. In mice, it has been shown that LPS endotoxemia increases pro-inflammatory cytokines such as IL-6 and TNF, detectable as early as 2 h and remaining elevated for 24 h [50]. Echocardiographic and myocardial strain measurements of cardiac function show a significant decline in cardiac function, cardiac output, and strain as early as 2 h, peaking at 12 h, and a significant decrease in heart rate. Systolic, diastolic, and mean arterial blood pressure measurements are all significantly depressed by 2 h and remain constant, whereas body temperature continuously declines [50]. Increases in systemic vascular resistance are evident at 6 h and peak at 12 h. Based on these functional studies and the expected half-life of LPS being approximately 12 h, we used this time point to study the effects of endotoxemia induced by LPS [51]. While we did not measure pro-inflammatory cytokines or cardiovascular responses in vivo, our prior study using the same LPS dose herein was sufficient to induce contractile dysfunction in ex vivo Langendorff-perfused heart preparations and in cardiac myocytes isolated from mice [30].

**Fig. 3:**
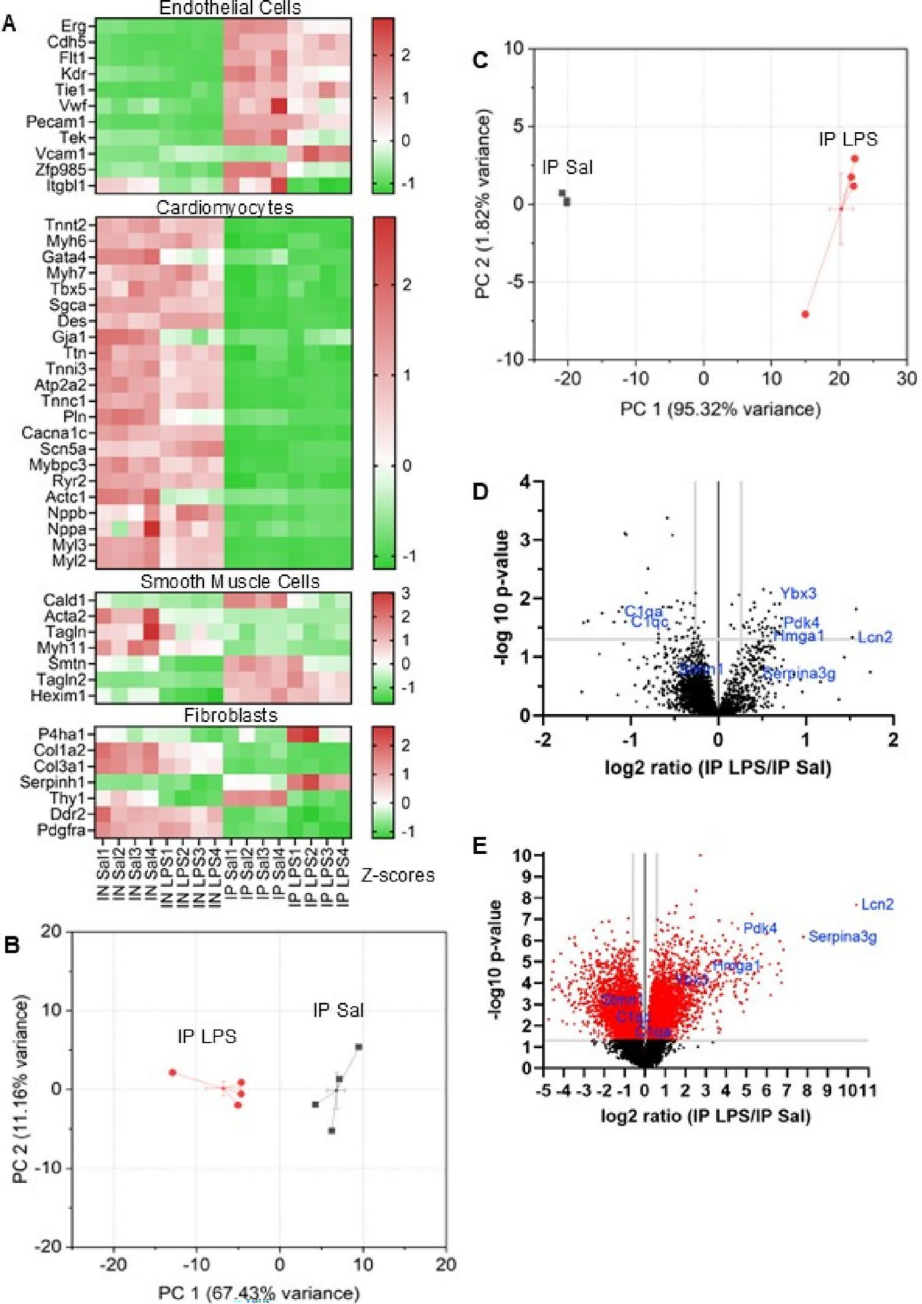
Multi-omic analysis of lipopolysaccharide (LPS) vs. saline (Sal) treatment in endothelial cells and whole heart homogenates. **A**, Heatmap of cell type-specific gene markers from RNA-Seq data indicating successful enrichment of endothelial cells in the immunoprecipitated (IP) fraction. The abbreviation IN represents input (whole heart homogenate) samples. **B**, Principal component (PC) analysis shows two distinct populations for the proteomic immunoprecipitated (IP) LPS *v* Sal analysis. Each point represents an individual biological replicate, and lines connect replicates within groups. Percent variance explained by each principal component is indicated on the axis. Error bars represent standard error, *n* = 4. **C**, Principal component (PC) analysis shows two distinct populations for the transcriptomic immunoprecipitated (IP) LPS vs. Sal analysis. Each point represents an individual biological replicate, and lines connect replicates within groups. Percent variance explained by each principal component is indicated on the axis. Error bars represent standard error, *n* = 4. **D**, Volcano plot of differential protein abundances between immunoprecipitated (IP) LPS vs. Sal, plotting -log10 p-values and Log2 ratio, *n* = 4. Threshold lines indicate – log10 p-value < 0.05 and a > 1.2-fold change. No data points in this analysis reached the predefined significance threshold (q-value < 0.05). **E**, Volcano plot of differential gene expression between immunoprecipitated (IP) LPS vs. Sal, plotting -log10 p-values and Log2 ratio, *n* = 4. Threshold lines indicate – log10 p-value < 0.05 and a > 1.5-fold change. Red data points indicate q-values < 0.05, corrected for false discovery rate using the two-stage step-up method of Benjamini, Krieger, and Yekutieli [40]

To characterize the immunoprecipitated protein and transcript pools enriched in the immunoprecipitated polysome fractions, we compared IP and IN datasets from saline- and LPS-treated samples (IP Sal vs. IN Sal and IP LPS vs. IN LPS). These comparisons revealed substantial differences in both protein abundances (affecting nearly half of the detected proteins) and transcript levels (over two-thirds), with clear separation in principal component analyses, suggesting distinct molecular profiles (Table 1, Fig. S1). Further evidence of effective endothelial cell enrichment was demonstrated by the selective enrichment of endothelial-specific transcripts in both IP groups (IP Sal and IP LPS), coupled with the depletion of transcripts from non-endothelial cell types, especially cardiomyocytes, which comprise the majority of heart tissue by volume [52] (Fig. 3A). Thus, effective EC enrichment allowed us to determine LPS-induced changes specifically in ECs while also allowing comparison to whole heart homogenate containing mixed cell populations.

**Table 1:** Summary of protein group and gene transcript identifications (ID) after filtering and mapping in ingenuity pathway analysis

Proteomics	Protein group total IDs	Protein groups *p*-value < 0.05	Protein groups q-value < 0.05	Protein groups up reg	Protein groups down reg
IP Sal v IN Sal	2,965	1,370	1,259	612:567	745:683
IP LPS v IN LPS	2,965	1,463	1,390	567:544	881:836
IP LPS v IP Sal	2,965	92	0	36:0	47:0
IN LPS v IN Sal	2.965	105	15	52:14	9:0

Transcriptomics	Gene total IDs	Gene IDs p-value < 0.05	Gene IDs q-value < 0.05	Gene IDs up reg	Gene IDs down reg
IP Sal v IN Sal	11,962	9,061	9,943	3,795:3,915	3,429:3,534
IP LPS v IN LPS	11,962	8,075	8,786	3,520:3.665	3,175:3,302
IP LPS v IP Sal	11,962	6,636	6,585	2,152:2,148	2,403:2,388
IN LPS v IN Sal	11,962	6,371	6,203	1,659:1,647	2,093:2,066

Proteomics + transcriptomics	Concordantly reg	Concordantly up reg	Concordantly down reg		
IP LPS v IP Sal	1,263	444	819		
IN LPS v IN Sal	1,518	702	816		

Proteomic data were filtered using thresholds of p-value and q-value < 0.05, and > 1.2-fold change for both up- and downregulated (Reg) protein groups. Transcriptomics data were filtered using thresholds of p-value and q-value < 0.05 and > 1.5-fold change in either direction (Tables S2 & S3). Concordantly up- or downregulated molecules across proteomic and transcriptomic datasets were identified based on directionality of log2 ratio, without applying p, q-value, or fold-change cutoffs (Tables S4 & S5). All concordant data are shown in Tables S4 (IP LPS v IP Sal) and S5 (IN LPS v IN Sal) under the concordant tabs within the Excel workbook. Statistical analysis was performed using an unpaired t-test (p-value) with an assumption of individual variance for each row. A false discovery rate (q-value) multiple comparison correction utilized Benjamini, Krieger, and Yekutieli’s two-stage step-up method [40]. *IP* immunoprecipitation, *Sal* saline, *LPS* lipopolysaccharide, *IN* input

### Effects of lipopolysaccharide endotoxemia on the endothelium

Vascular inflammation is central to endotoxemia, and while immune cells produce cytokines, the endothelium is activated to modulate the inflammatory response. Prolonged endothelial cell activation induced by inflammatory cytokine secretion can lead to endothelial dysfunction and apoptosis, thereby affecting vascular permeability, impairing blood flow, and potentially causing organ damage [53]. Principal component analysis of the LPS vs. saline-treated samples in both EC fractions and whole heart homogenates showed clear separation between conditions (Fig. 3B, C, Fig. S1). As an initial step in analyzing the EC proteomics data, we identified significantly altered gene sets - activated or suppressed - based on the directionality of protein abundance and a p-adjusted value of ≤ 0.05 (Fig. 2S). This analysis revealed that the functions associated with the upregulated proteins included mRNA stabilization, gene silencing, regulation of post-transcriptional events, and the immune response following LPS treatment. The functions related to the downregulated EC proteins were associated with cellular respiration and metabolism in response to LPS. Thus, the changes in protein abundance observed at 12 h post-LPS treatment suggest a coordinated response aimed at assembling protein networks that regulate co-translational processes, potentially as a compensatory mechanism against vascular inflammation.

**Fig. 4:**
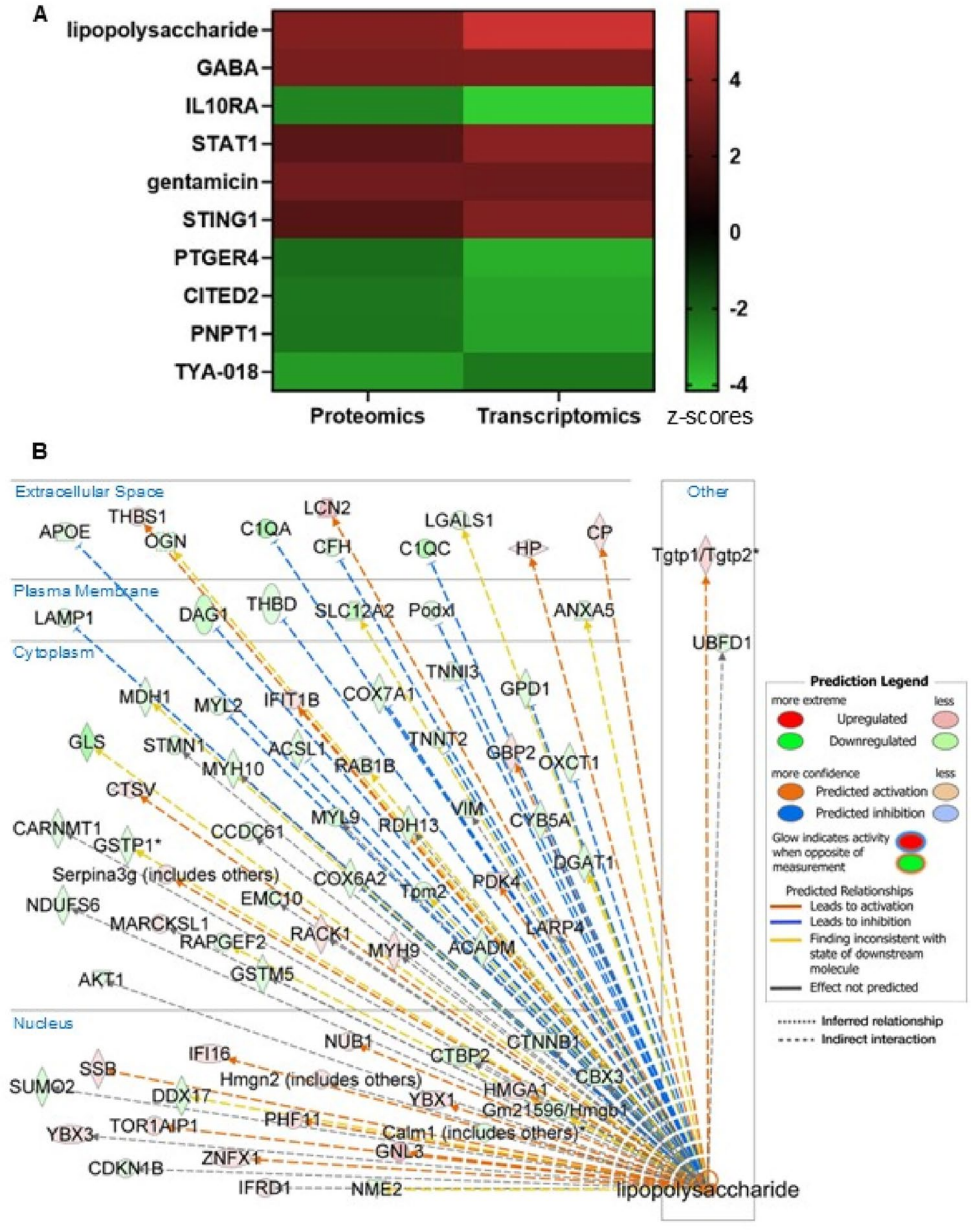
Ingenuity pathway analysis of upstream regulators. **A**, Heatmap of the top 10 upstream regulator pathways found and ranked based on a z-score. Upregulated pathways are shown in shades of red, and downregulated pathways are shown in shades of green. Data are derived from the Log2 ratio of IP LPS/IP Sal proteomic and transcriptomic concordant data. The predicted top upstream regulator in endothelial-enriched cells treated with lipopolysaccharide is lipopolysaccharide. **B**, Subcellular mapping of lipopolysaccharide-associated upstream regulators based on proteomic data. This diagram displays upstream regulators of the LPS response, organized by subcellular localization, with gene names and relative abundance derived from the Log_2_ ratio of IP LPS/IP Sal proteomic concordant data. Regulators are mapped to the nucleus, cytoplasm, plasma membrane, and extracellular space, with the cytoplasm containing the highest number of altered components. Upregulated genes are shown in shades of red, downregulated genes in shades of green, while predicted activation and inhibition are represented by orange and blue, respectively, as indicated in the prediction legend to the right

To gain deeper insight, we aligned the proteomic and transcriptomic datasets to examine the regulation of 2,965 proteins (Table 1 and Table S2) and 11,962 transcripts (Table 1 and Table S3) in response to LPS treatment, based on concordant or discordant expression changes. Our analysis revealed that 1,263 protein groups were concordantly regulated in the endothelial-enriched pool compared to 1,518 in whole heart lysates (Table 1, Tables S3 & S4). We plotted them in volcano plots to illustrate the concordant effects of LPS treatment across the proteomic and transcriptomic datasets. We labeled the proteins and genes involved with the LPS pathway of upstream regulators in the volcano plots (Fig. 3D-E, Fig. S3). Although the overall proteomic abundances of the IP LPS/IP Sal comparison of ECs did not reach a q-value cutoff below 0.05, the majority of the LPS pathway of upstream regulators (lipocalin-2, pyruvate dehydrogenase kinase 4, B-box binding protein 3, high mobility group AT-hook 1, complement C1q subunits a and c chains) did reach a p-value cutoff below 0.05 and a log2 ratio ≥ 20% difference (Fig. 3D). The proteomic data were aligned with the transcriptomic results that met the significance threshold (0.05 q-value) and exhibited a log2 fold change of ≥ 50% difference (Fig. 3E). The IP and IN (whole homogenate) datasets were analyzed using IPA (Ingenuity Pathway Analysis) to enable unbiased identification and prediction of the pathways and regulators associated with LPS-induced endotoxemia. As expected, lipopolysaccharide was identified as the top upstream regulator based on the concordant changes in both the proteomic and transcriptomic datasets (Fig. 4A, Fig. S4). This prediction was supported by over 50 concordantly regulated proteins and transcripts, which traced the signal cascade back to LPS in both endothelial-enriched (IP LPS vs. IP Sal) and whole homogenate IN (LPS vs. IN Sal) samples. The independent identification of LPS as a dominant upstream driver confirms the biological relevance of the observed responses and validates the utility of our integrated co-translational profiling approach for predicting disease-relevant regulatory pathways in vivo (Fig. 4B, Fig. S4).

**Fig. 5:**
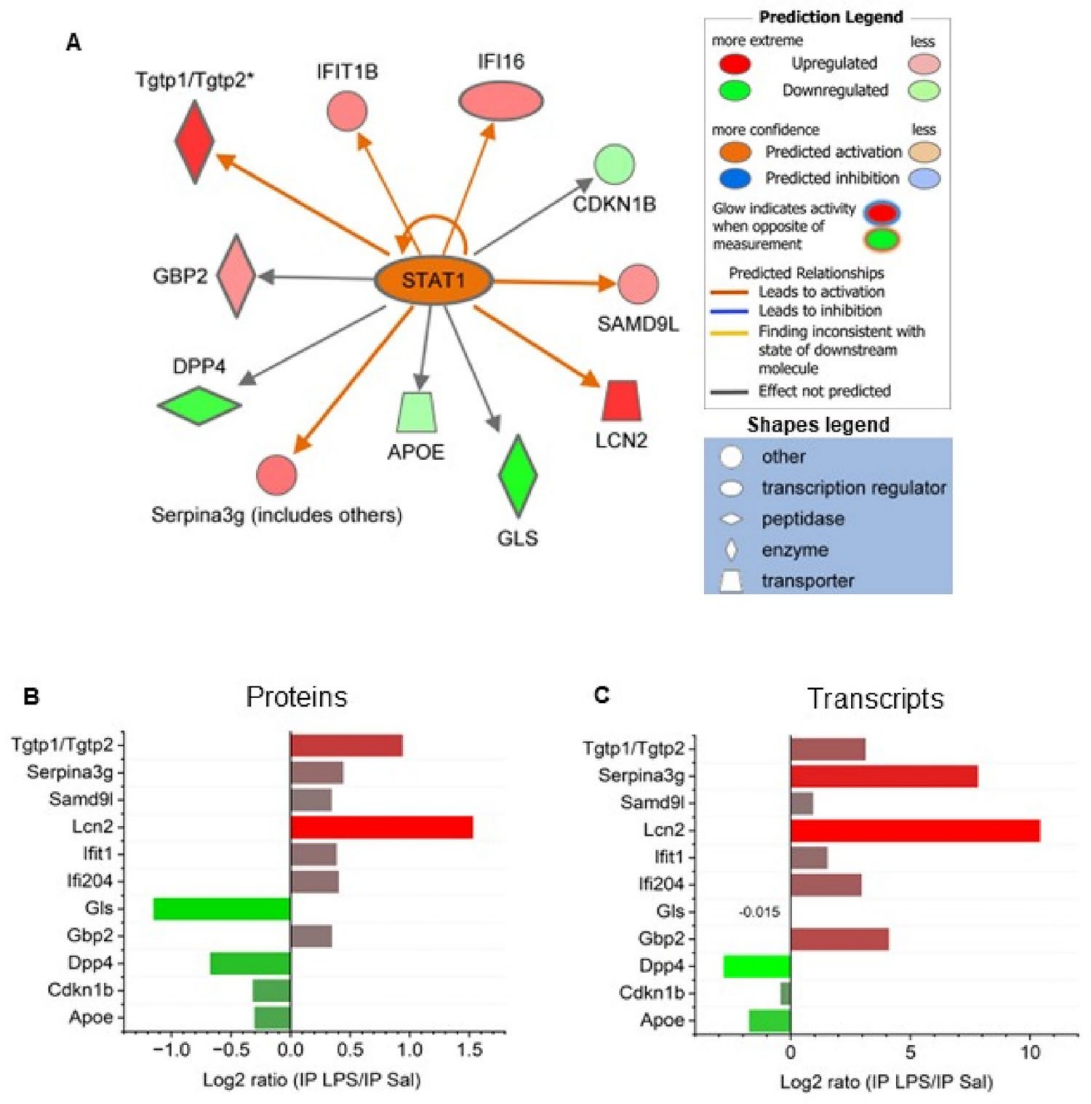
Predicted activation of STAT1 based on Ingenuity Pathway Analysis of concordant (proteomic and transcriptomic) data from Fig. 4**A**. Data are derived from the Log_2_ ratio of IP LPS/IP Sal proteomic and transcriptomic concordant data. **A**, An organic layout showing the proteomic STAT1 upstream regulator pathway for IP LPS vs. IP Sal comparison. Upregulated genes are shown in shades of red, downregulated genes in shades of green, while predicted activation and inhibition are represented by orange and blue, respectively, as indicated in the prediction legend to the right. The shapes legend, also to the right, indicates the protein family. **B**, Bar graph with color mapping of STAT1 upstream regulator pathway components and their relative abundance from the proteomics dataset in panel **A**. Upregulated protein abundance is shown in shades of red, while downregulated protein abundance is shown in shades of green, based on log₂ ratio values. **C**, Bar graph with color mapping of STAT1 upstream regulator pathway components and their relative expression from the transcriptomics dataset in panel **A**. Upregulated protein abundance is shown in shades of red, while downregulated protein abundance is shown in shades of green, based on log_2_ ratio values. Note: STAT1 is predicted to be activated with LPS treatment

In addition to LPS, both STAT1 and STING1 were identified in the group of top 10 upstream regulators and were predicted to be activated, contributing to the concordant regulation of transcript and protein expression (Fig. 4A, Fig. S4). STAT1 is of interest due to its involvement in inflammation as a response to an infection via the JAK/STAT signaling cascade. Within our concordant dataset, STAT1 was predicted to regulate the expression of multiple transcripts and proteins, most of which are associated with the inflammatory response to endotoxemia (Fig. 5). Notably, the most highly upregulated gene and protein was lipocalin-2 (Lcn2) (Fig. 5B-C), a known antimicrobial factor that limits bacterial growth by sequestering iron [54].

Finally, while our EC-specific co-translational profiling focused primarily on the concordant changes within the polysome pool associated with vascular inflammation, nearly half of the identified proteins (1,362 of 2,965) in ECs were discordantly regulated (Table 2, Tables S4 &S5). Among these, comparison of discordant expression, specifically cases where proteins were downregulated despite upregulated transcripts, revealed that such transcripts were markedly more frequent in the ECs (79) than in the whole heart homogenates (4). To determine which cellular processes were most likely affected, we examined the protein networks associated with discordantly regulated proteins enriched in the endothelium (Table S4). KEGG pathway enrichment analysis revealed that proteins involved in glucose metabolism via glycolysis were overrepresented in this discordant group (Fig. S5). Specifically, the expression of glycolytic proteins such as Hexokinase-1 (HK-1), Pyruvate kinase (PKM), Fructose-1,6-bisphosphatase isozyme 2 (FBP2), Phosphoglycerate kinase 1 (PGK1), and Glyceraldehyde-3-phosphate dehydrogenase (GAPDH) was repressed, while their respective transcripts were increased (Table S4). Given their relatively low mitochondrial content, ECs primarily rely on glycolysis for energy production [55]. HK1 mediates the initial step of glycolysis by phosphorylating D-glucose to D-glucose 6-phosphate, a key precursor not only for glycolysis but also for biosynthetic pathways, such as the pentose phosphate pathway (PPP). The intracellular localization of HK1 is a critical determinant of glucose metabolism and metabolite generation, as demonstrated in macrophages [56]. While metabolic reprogramming, characterized by enhanced glycolysis and reduced oxidative phosphorylation, occurs in immune cells and ECs during inflammation, EC metabolism appears somewhat similar across vascular beds [57]. Mice treated with the nonmetabolizable glucose analog 2-deoxy-D-glucose, a competitive inhibitor of glycolysis to restrict glycolytic flux, show improved cardiovascular function and mortality in polymicrobial-induced infection [58]. While not explicitly studied here, the translational repression of glycolysis-associated proteins may function as an adaptive mechanism to limit the endothelial proinflammatory phenotype, suggesting additional levels of co-translational regulation in the endothelium following LPS treatment. These may include processes such as removing and degrading defective or damaged mRNA transcripts that can trigger ribosomal stalling, or the misfolding of nascent proteins targeted for degradation by the ubiquitin-proteasome system. Alternatively, this pattern may reflect a temporal phase in which transcripts are loaded onto polysomes but active translation has not yet occurred, or in which protein abundance remains below the detection threshold.

**Table 2:** Summary of discordant protein group and gene transcript identifications (IDs) after filtering, exporting ingenuity pathway analysis mapping, and alignment of datasets in excel

Discordant data IDs	Log 2 ratio (IP LPS v IP Sal)	Log2 ratio (IN LPS v IN Sal)
Protein up reg IDs	59	20
Transcript down reg IDs	85	192
Protein up reg and transcript down reg IDs	22	7
Protein down reg IDs	270	8
Transcript up reg IDs	422	64
Protein down reg and transcript up reg IDs	79	4
Total IDs	1,357	1,104

Protein groups were filtered based on 1.2-fold change in the up- and down-regulated (Reg) abundance, while transcriptomic data were filtered based on s 1.5-fold change in expression. Discordant regulation was defined by directionality of the log2 ratio (1.2 and 1.5 fold change, respectively) and had no associated p- or q-value. All discordant data are available in Tables S4 (IP LPS v IP Sal) and S5 (IN LPS v IN Sal) under the “Discordant” tabs of the Excel workbook. The light gray sections of the table represent aligned transcript-protein pairs showing discordant data (also available in Tables S4, S5 under dedicated Excel tabs). These reflect disruptions in the expected flow of the central dogma, where transcript and protein levels do not correlate. In contrast, the white sections display discordant proteins or transcripts analyzed independently. A notable distinction is observed between sample types: whole heart homogenates exhibited 4 vs. 7 discordant features, while the endothelial-enriched fractions showed 79 vs. 22, more than a 3-fold difference, suggesting perturbed endothelial cell-specific translation in response to LPS. *IP* immunoprecipitation, *Sal* saline, *LPS* lipopolysaccharide, *IN* input

The initial focus of this proof-of-concept study was to establish this approach in the context of acute perturbation. While the present study has limitations, it also presents equally important possibilities for further development and adaptation. Limitations include the lack of temporal and LPS dose-response analyses, which could provide insight into the potential mechanistic steps involved in regulating this discordant dataset. Identifying these targets in the cell could open the possibility of adapting novel reporters specifically developed for the analysis of ribosomal stalling in a target-specific, physiological-response context in vivo [59]. The shortage of investigation into sex- and age-related endothelial responses to endotoxemia-induced vascular inflammation, which are clinically relevant. Although the mechanistic basis of sex differences remains unclear, several factors, such as estrogen’s vasoprotective and immunomodulatory actions, play a role. Estrogen increases the synthesis and release of endothelial-derived vasodilators such as nitric oxide and prostacyclin, and modulates the expression of pro-inflammatory mediators, such as C-reactive protein, and cytokine-induced neutrophil chemoattractant (CINC)−2β [60–63]. In the context of aging, the inflammatory response does not appear to be exacerbated, whereas the endothelial response is in response to LPS-induced endotoxemia. Endothelial-mediated vasodilation is impaired, and increases in biomarkers associated with endothelial glycocalyx injury and permeability were elevated sooner in older mice [64]. Finally, while we did not examine or correlate changes in our “functional translatome” with physiological function in vivo, we aligned our protocol with the prior study, which used the ex vivo technique of retrograde perfusion of an isolated heart. However, our multi-omics approach to investigate cell-specific co-translational regulation by modifying the translating ribosome affinity purification (TRAP) approach in vivo, lends itself to future cardiovascular-related studies, by breeding the RiboTag (*Rpl22*^*HA*/+^) line with different cell-type specific Cre-driver lines, to support comparative analysis of in vivo or ex vivo function and cellular crosstalk in a number of different cardiovascular developmental and disease models. Consequently, this study highlights the utility of our “functional translatomics” discovery approach in enabling deeper, predictive analysis of disease pathways and interventions based on co-translational regulation. This strategy integrates cell-type-specific proteomic and transcriptomic data to identify co-translational regulatory events and offers a clearer understanding of how regulatory networks coordinate protein expression at the interface of transcription and translation. Furthermore, this approach advances our understanding of translational quality control checkpoints involved in the production of functional proteins and facilitates the identification of concordantly and discordantly regulated signaling networks during the emergence of newly activated pathways.

## Supplementary Information

Below is the link to the electronic supplementary material.

Supplementary Material 1

Supplementary Material 2

Supplementary Material 3

Supplementary Material 4

Supplementary Material 5

Supplementary Material 6

## Data Availability

The proteomics dataset is available via the MassIVE data repository, a full member of the ProteomeXchange Consortium ([Welcome to MassIVE](https:/massive.ucsd.edu/ProteoSAFe/static/massive.jsp)). The MassIVE dataset identifier is MSV000095131. The transcriptomic dataset is available via the NCBI Gene Expression Omnibus repository at [https://www.ncbi.nlm.nih.gov/geo](https:/www.ncbi.nlm.nih.gov/geo) under the accession number GSE273368. All other data is contained within this published article (supplementary files).

## Abbreviations

IP: Immunoprecipitation
RiboTag_EC_: Cdh5CreERT2
LPS: Lipopolysaccharide
Ribo-Seq: Ribosomal proteomics and profiling
RiboTag: Ribosomal protein epitope-tagged transgenic reporter mouse
IN: Input
Sal: Saline
TEAB: Triethylammonium bicarbonate
TMT: Tandem mass tag
PBST: Phosphate-buffered saline with tween-20
TBST: Tris-buffer saline with tween-20
PCA: Principal component analysis
IPA: Ingenuity pathway analysis
GSEA: Gene set enrichment analysis
EC: Endothelial cell
TRAP: Translating ribosome affinity purification
RFPs: Ribosome footprints
HA: Hemagglutinin
PKM: Pyruvate kinase
HK-1: Hexokinase-1
FBP2: Fructose-1,6-bisphosphatase isozyme 2
PGK1: Phosphoglycerate kinase 1
GAPDH: Glyceraldehyde-3-phosphate dehydrogenase
PPP: Pentose phosphate pathway
FDR: False discovery rate
