# Supplementary material for: Co-translational profiling in the cardiac endothelium in response to LPS-induced inflammation in female mice in vivo: a proof-of-concept approach
Source: Mol Cell Biochem. 2026 Apr 28;481(6):2381–98. doi: 10.1007/s11010-026-05551-9 (PMC13279726; doi:10.1007/s11010-026-05551-9)
Supplement: Supplementary file 6 — Supplementary Material 6 [file 11010_2026_5551_MOESM6_ESM.docx]

**Supporting information for:**

**Article title:**

**Co-translational profiling in the cardiac endothelium in response to LPS-induced inflammation in female mice *in vivo*: a proof-of-concept approach**

**Authors:**

Chad M. Warren^1^, Bhairavi Swaminathan^1^, Paulina Langa^1^, Stephanie R. Villa^1^, Walter C. Thompson^1^, Magdalena Chrzanowska^2,3,4,5^, Jan K. Kitajewski^1,6,7^, R. John Solaro^1,6^, Beata M. Wolska^1,6,8^, Paul H. Goldspink^1,6*^.

^1^Department of Physiology and Biophysics, University of Illinois, Chicago, IL, USA.

^2^Versiti Blood Research Institute, Milwaukee, WI, USA.

^3^Department of Pharmacology and Toxicology, Medical College of Wisconsin, Milwaukee, WI, USA.

^4^Cancer Center, Medical College of Wisconsin, Milwaukee, WI, USA.

^5^Cardiovascular Center, Medical College of Wisconsin, Milwaukee, WI, USA.

^6^Center for Cardiovascular Research, University of Illinois, Chicago, IL, USA.

^7^University of Illinois Cancer Center, University of Illinois, Chicago, IL, USA.

^8^Department of Medicine, Division of Cardiology, University of Illinois, Chicago, IL, USA.

The supplemental information includes Figures S1 to S5 and supporting table descriptions

**Supplemental Figures**


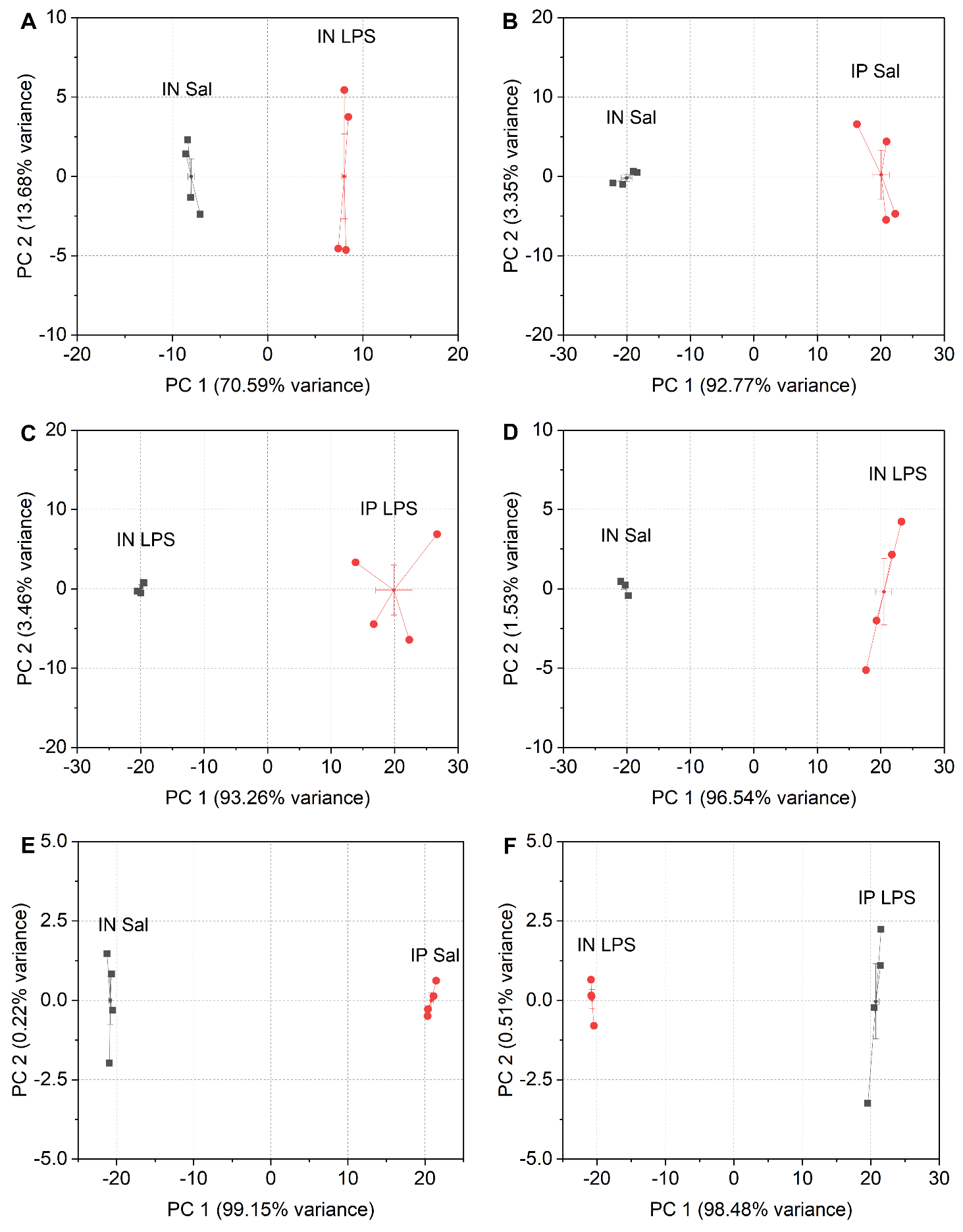


**Figure S1.** Principal component analysis (PCA) indicates distinct populations for multiple binary comparisons in proteomic and transcriptomic data. ***A-C****,* PCA analysis of proteomic data with error bars representing standard error and n=4. ***D-F****,* PCA analysis of transcriptomic data with error bars representing standard error and n=4. Groups were IN Sal, input saline; IN LPS, input lipopolysaccharide; IP Sal, immunoprecipitated saline; IP LPS, immunoprecipitated lipopolysaccharide. Each point represents an individual biological replicate, and lines connect replicates with groups. The percent variance explained by each principal component is indicated on the axis.

**
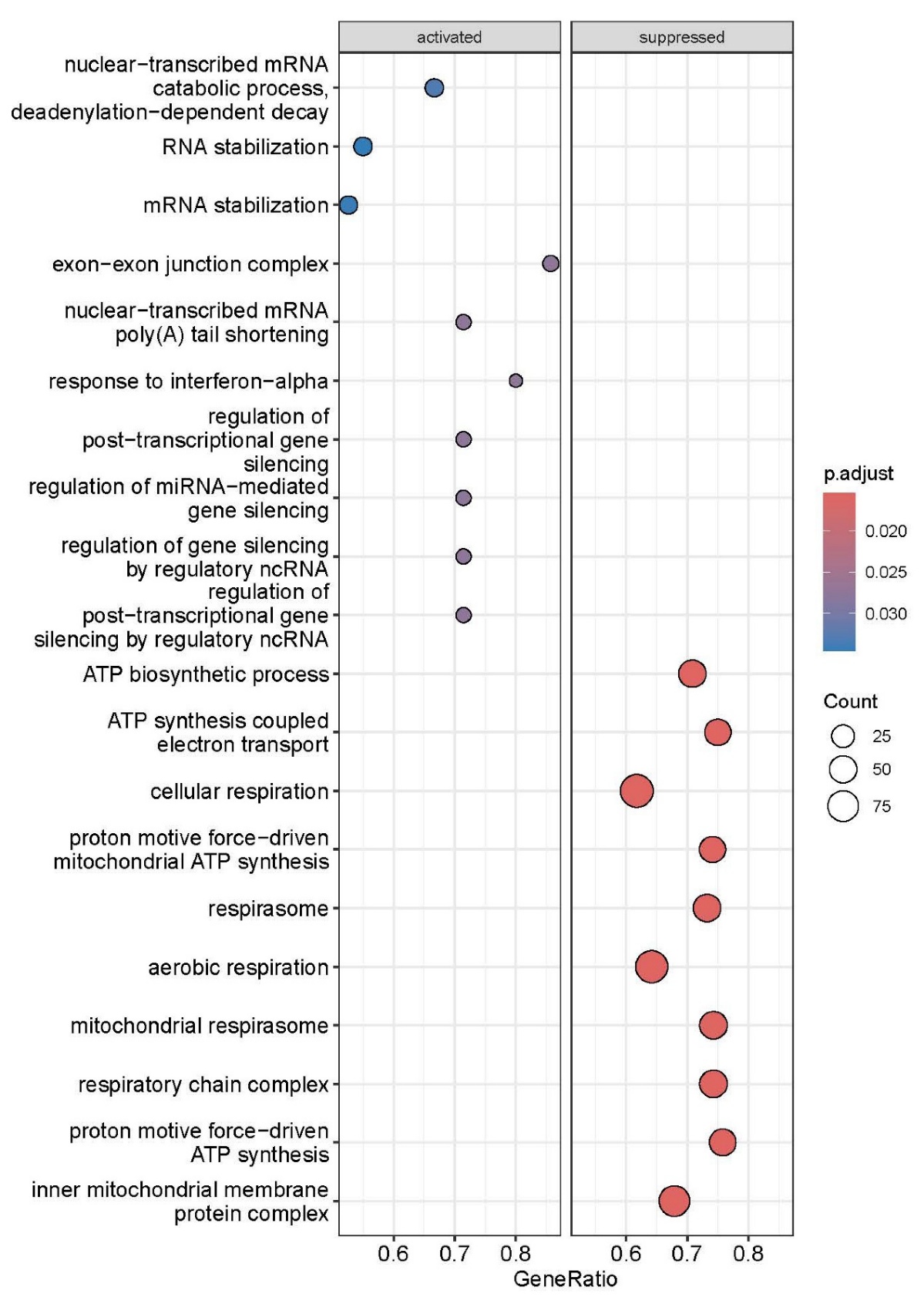
**

**Figure S2**. Dot plot showing the top 20 significant pathways that are enriched (left) or suppressed (right) in ECs after LPS treatment. Gene Set Enrichment Analysis (GSEA) was generated using ClusterProfiler from the IP LPS *vs.* IP Sal proteomic data. The size of the dots represents the number of proteins in each category, while the color of the dots represents the p-adjusted value, and the GeneRatio is the percentage of proteins in the leading-edge subset that actively contributes to the enrichment score.


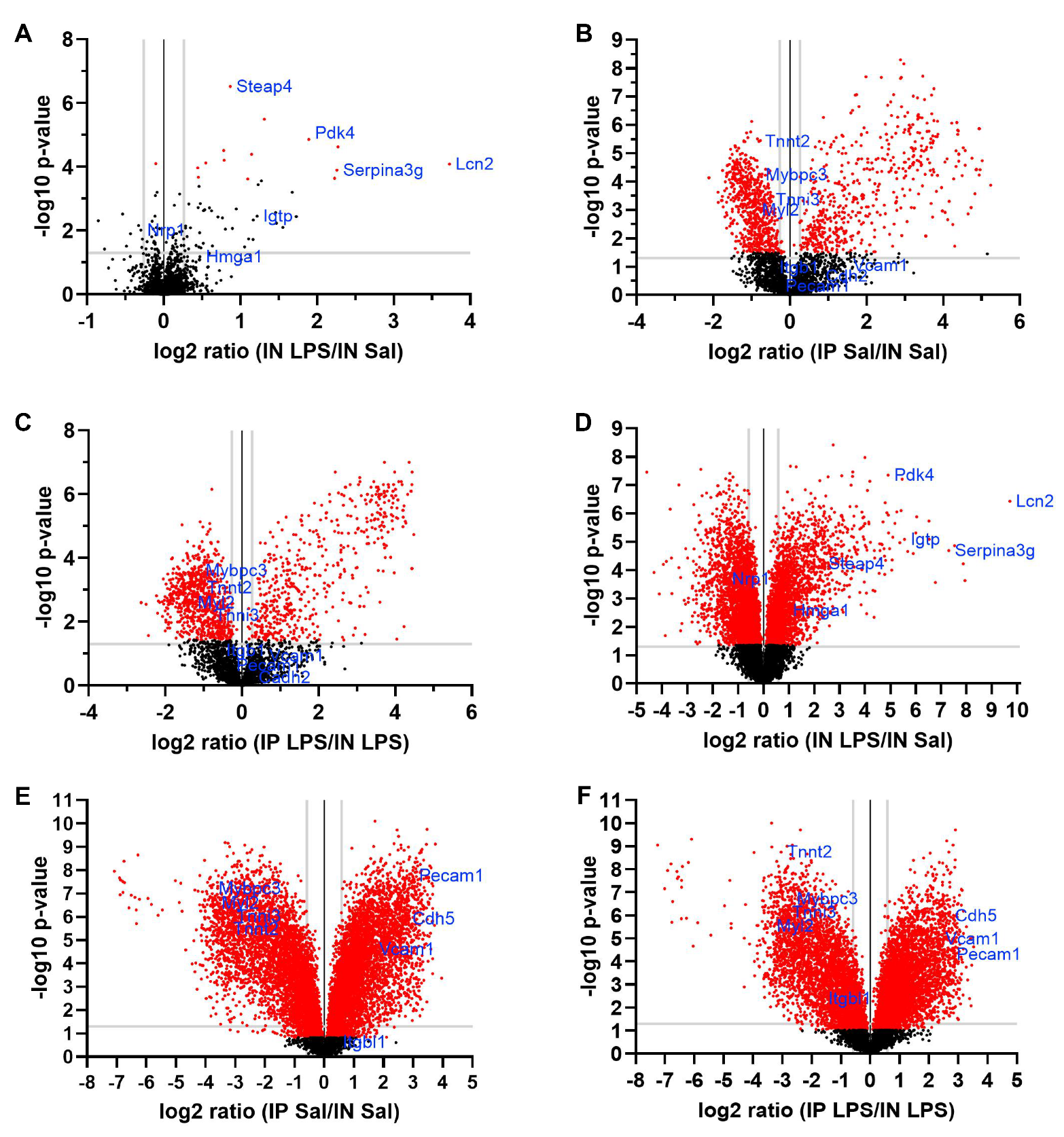


**Figure S3.** Volcano plots of differential abundance and expression across multiple binary comparisons in proteomic and transcriptomic datasets. ***A-C****,* Volcano plots of differential protein abundances, plotting -log10 p-values and Log2 ratio, n=4. Threshold lines indicate – log10 p-value < 0.05 and a > 1.2-fold change. ***D-F****,* Volcano plots of differential gene expression, plotting -log10 p-values and Log2 ratio, n=4. Threshold lines indicate – log10 p-value < 0.05 and a > 1.5-fold change. Groups were IN Sal, input saline; IN LPS, input lipopolysaccharide; IP Sal, immunoprecipitated saline; IP LPS, immunoprecipitated lipopolysaccharide. Red data points indicate a q-value < 0.05, based on a false discovery rate multiple-comparison correction using Benjamini, Krieger, and Yekutieli's two-stage step-up method.

**
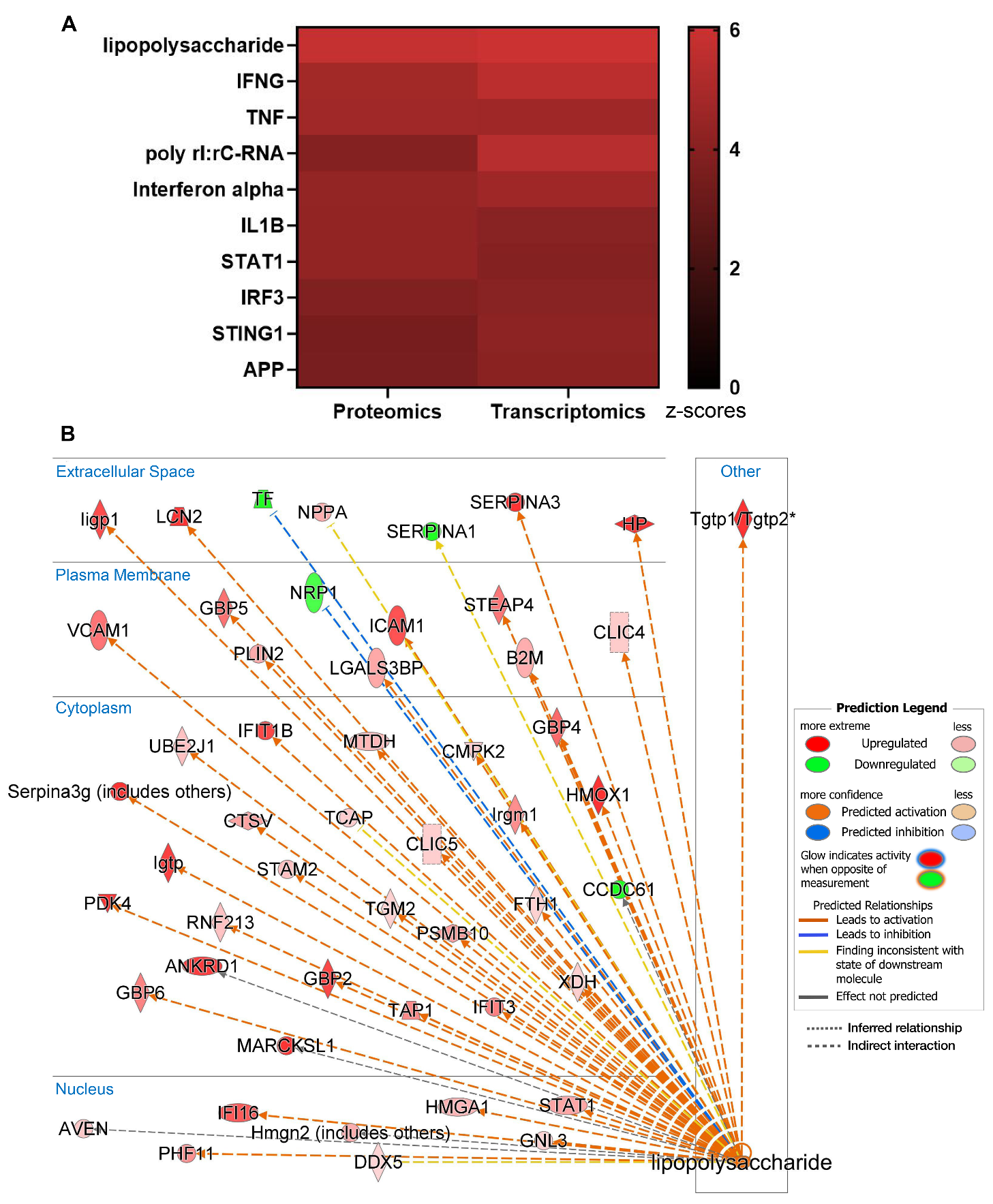
**

**Figure S4.** Ingenuity pathway analysis of concordantly regulated proteomic and transcriptomic datasets in whole-heart homogenates (IN LPS vs. IN Sal) showed that the predicted top upstream regulator was lipopolysaccharide. ***A****,* Heatmap of the top 10 upstream regulators in both proteomic and transcriptomic data, ranked based on a z-score. ***B****,* A diagrammatic prediction of the lipopolysaccharide subcellular pathway, with protein names and relative abundance depicted. Data are derived from the Log_2_ ratio of IN LPS/IN Sal proteomic concordant data. The upstream regulators are further separated into subcellular compartments, with the cytoplasm showing the greatest alteration. Upregulated proteins are shown in shades of red, downregulated proteins in shades of green, as indicated in the prediction legend to the right.


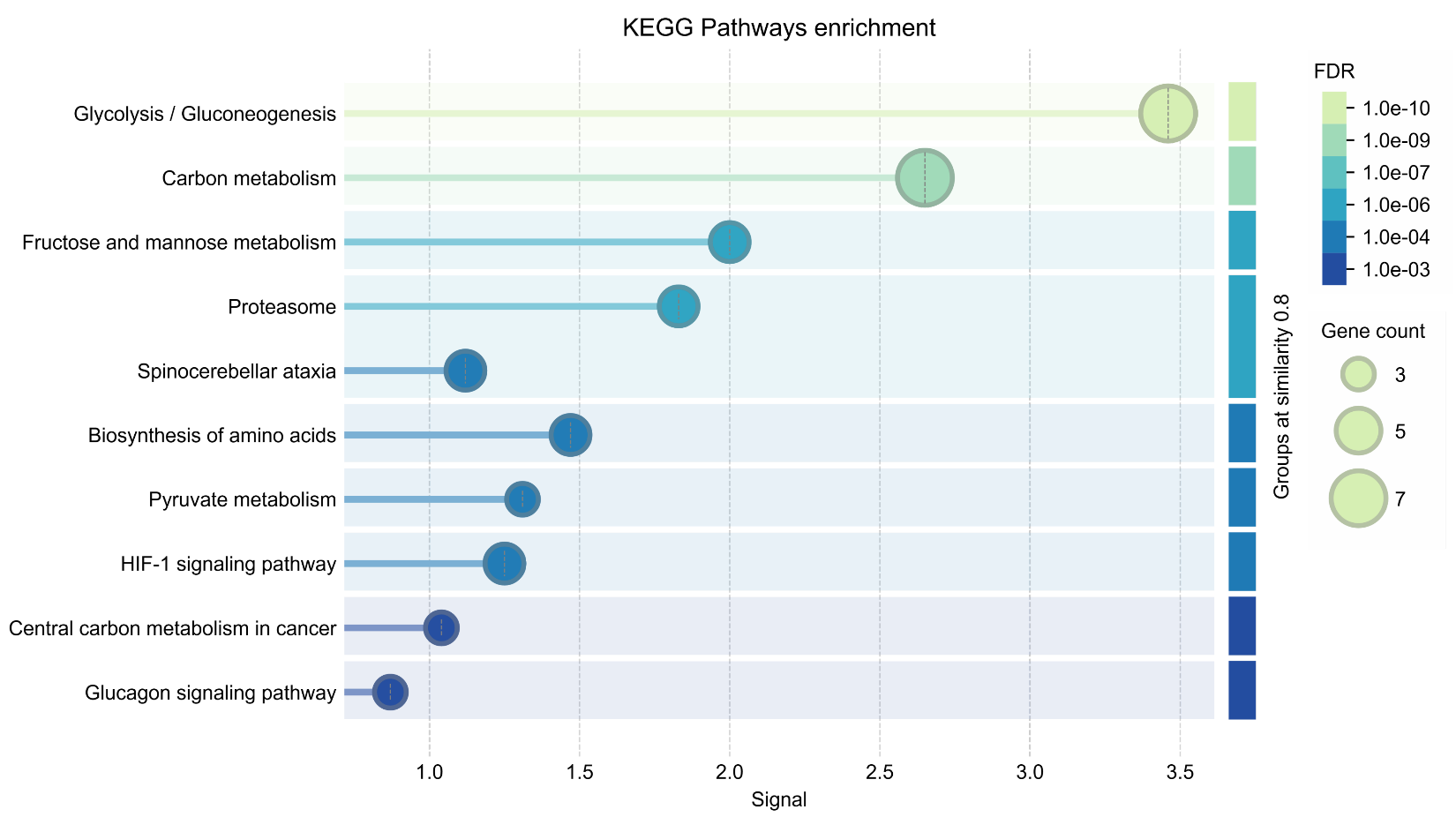


**Figure S5.** String-db.org was used to generate KEGG pathway enrichment for IP LPS vs. IP Sal discordant data (under a “Creative Commons BY 4.0” license), where protein abundance was down while transcript abundance was upregulated. The top 10 pathways are shown from the top cluster (+ Glucose catabolism), with their associated false discovery rate (FDR) color-coded and gene count indicated by the circle size. The signal on the x-axis is a weighted harmonic mean of the observed/expected ratio and the -log FDR, which aims to provide a more intuitive ordering of the enriched pathways. The terms were grouped by a default similarity score of > 0.8.

Supplemental Table Descriptions

**Table S1.** Ribosomal proteins that were identified in the IP fractions. 75 of the 80 predicted ribosomal proteins were identified in the IP LPS / IP Sal comparison, all with positive log-ratios (IP LPS / IP Sal), suggesting successful enrichment of endothelial cells, as the HA tag was attached to ribosomal protein L22.

**Table S2.** The complete quantitative proteomics data for all 4 binary comparisons are in 4 separate Excel workbook tabs. The 4 tabs are IP LPS v IP Sal, IN LPS v IN Sal, IP Sal v IN Sal, and IP LPS v IN LPS.

**Table S3.** The complete quantitative transcriptomics (RNA-seq) data for all 4 binary comparisons are in 4 separate Excel workbook tabs. The 4 tabs are IP LPS v IP Sal, IN LPS v IN Sal, IP Sal v IN Sal, and IP LPS v IN LPS.

**Table S4.** The concordant and discordant data for the IP LPS v IP Sal comparison. There are 4 discrete Excel workbook tabs with concordant, discordant (all), Discordant P-up T-down, and Discordant P-down T-up.

**Table S5.** The concordant and discordant data for the IN LPS v IN Sal comparison. There are 4 discrete Excel workbook tabs with concordant, discordant (all), Discordant P-up T-down, and Discordant P-down T-up.
